# Common and distinct regulation of human and mouse brown and beige adipose tissues: a promising therapeutic target for obesity

**DOI:** 10.1007/s13238-017-0378-6

**Published:** 2017-02-20

**Authors:** Xuejiao Liu, Christopher Cervantes, Feng Liu

**Affiliations:** 1 Department of Metabolism and Endocrinology, Metabolic Syndrome Research Center of Central South University, The Second Xiangya Hospital, Central South University, Changsha, 410011 China; 2 Department of Pharmacology, University of Texas Health Science Center at San Antonio, San Antonio, TX 78229 USA

**Keywords:** human brown adipose tissue, energy metabolism, obesity

## Abstract

Obesity, which underlies various metabolic and cardiovascular diseases, is a growing public health challenge for which established therapies are inadequate. Given the current obesity epidemic, there is a pressing need for more novel therapeutic strategies that will help adult individuals to manage their weight. One promising therapeutic intervention for reducing obesity is to enhance energy expenditure. Investigations into human brown fat and the recently discovered beige/brite fat have galvanized intense research efforts during the past decade because of their pivotal roles in energy dissipation. In this review, we summarize the evolution of human brown adipose tissue (hBAT) research and discuss new *in vivo* methodologies for evaluating energy expenditure in patients. We highlight the differences between human and mouse BAT by integrating and comparing their cellular morphology, function, and gene expression profiles. Although great advances in hBAT biology have been achieved in the past decade, more cellular models are needed to acquire a better understanding of adipose-specific processes and molecular mechanisms. Thus, this review also describes the development of a human brown fat cell line, which could provide promising mechanistic insights into hBAT function, signal transduction, and development. Finally, we focus on the therapeutic potential and current limitations of hBAT as an anti-glycemic, anti-lipidemic, and weight loss-inducing ‘metabolic panacea’.

## INTRODUCTION

Throughout human history, cold weather and famine were threats to survival. To ward off these threats, brown adipose tissue (BAT) and white adipose tissue (WAT) were evolutionarily selected. BAT dissipates energy to keep us warm, while WAT stores excess energy to be spent during times of food deficiency. These two complementary fat depots work to maintain energy balance. However, industrialization and technological advancements in developed societies have broken this balance due to an increase in sedentary occupations, overabundance of food, lack of physical activity, and warm shelter, causing obesity rates to skyrocket as more countries move from being undeveloped to developed. As the World Health Organization (WHO) reported in June 2016, worldwide obesity has more than doubled since 1980 (World Health Organization Fact Sheets: Obesity and Overweight, 2016). In 2014, 39% of adults aged 18 years and older were overweight, and 13% of them were obese (World Health Organization Fact Sheets: Obesity and Overweight, 2016). Most of the world’s population lives in countries where a high body mass index kills more people than a low one (World Health Organization Fact Sheets: Obesity and Overweight, 2016). In other words, food shortages have not been as deadly as food surpluses. According to the data, obesity, which is associated with type 2 diabetes, polycystic ovarian syndrome, hypertension, cardiovascular disease, etc., is the major public health challenge of the 21st century (Eckel et al., 2010). Thus, therapeutic approaches that prevent and/or reverse obesity deserve further exploration.

Brown adipocytes, which are rich in mitochondria, are different from white adipocytes because they consume energy more efficiently. Indeed, increased energy expenditure would be expected to reverse and/or prevent obesity (Hall et al., 2011). Given that active BAT with energy dissipation function has been confirmed in a number of human adults (Nedergaard et al., 2007; Cypess et al., 2009; Saito et al., 2009; van Marken Lichtenbelt et al., 2009), research interests into human BAT as a means of controlling obesity by diverting excess fat into heat has greatly intensified and represents a novel therapeutic approach for combatting this deadly, metabolic disease.

## THE EVOLUTION OF HUMAN BAT RESEARCH

In 1902, Shinkishi Hatai first reported in a human embryonic development study that BAT is located beneath the sternocleidomastoid and trapezius muscles and runs laterally and parallel to the neck (Hatai, 1902). During human infancy, heat is generated by BAT via non-shivering thermogenesis, which is the main defense our bodies employ against cold environments (Aherne and Hull, 1966). With increasing age, human brown adipose tissue (hBAT) was thought to diminish and ultimately vanish. It was not until his 1972 necropsy study that Heaton found human BAT persists throughout later stages of life, located at specific anatomical locations including around the kidneys, suprarenals and aorta, and in the neck and mediastinum (Heaton, 1972). In the 1980s, Huttunen et al. found that a large proportion of fat cells isolated from the neck contained potent mitochondrial enzymes in adult individuals exposed to cold (Huttunen et al., 1981). No active BAT had been identified in human adults prior to these discoveries because *in vivo* research into hBAT was severely hampered by a lack of methodologies that could accurately pinpoint its location.

In the mid-1990s, the emergence of positron emission tomography (PET) using the glucose tracer, 2-[18F]-fluoro-D-2-deoxy-D-glucose (FDG), combined with computer tomography (CT) renewed interest in hBAT and its role in energy expenditure (Cypess et al., 2009; Saito et al., 2009; van Marken Lichtenbelt et al., 2009). Just as in malignancy, hyper-metabolic hBAT has a high FDG uptake rate that can be detected by the minimally-invasive PET-CT technique *in vivo*. More recently, it has been discovered that a significant number of adult humans have active BAT that can be detected by PET-CT (Cypess et al., 2009; Saito et al., 2009; van Marken Lichtenbelt et al., 2009). However, PET-CT is not sensitive to BAT in the thermoneutral state because it depends on tissue glucose utilization (Chen et al., 2013). In recent years, magnetic resonance imaging (MRI) and functional (f)MRI, which detect changes associated with blood flow, have been used to assess hBAT volume and monitor responses to certain stimuli. MRI distinguishes between BAT and WAT according to three physiological criteria: BAT has a higher water-to-fat ratio, a higher density of mitochondria, and more blood vessels than WAT (Chen et al., 2013). According to imaging examinations, the main BAT depots are in the supraclavicular and neck regions with some additional paravertebral, mediastinal, para-aortic, and suprarenal localization (but not the interscapular region) (Nedergaard et al., 2007), which coincide with former anatomical results.

Great advances about hBAT biology have emerged from imaging methods in recent years, and many of the achievements concur with those of murine BAT. Based on a higher FDG uptake, PET-CT scans showed that cold exposure significantly enhances hBAT activation (Saito et al., 2009; van Marken Lichtenbelt et al., 2009). Gender is another key determinant of BAT mass and activity. In general, women contain more BAT than men, while BAT distributions are similar across sexes (Cypess et al., 2009). Moreover, BAT volume and cold-activated FDG uptake are inversely correlated with age, beta-blocker use, body-mass index, body fat, and visceral fat (Nedergaard et al., 2007; Cypess et al., 2009; Saito et al., 2009). Thus, BAT is more abundant in younger and leaner individuals and decreased by long-term beta-blocker use. Altogether, these novel approaches have significantly advanced our understanding about hBAT localization and regulatory function and have provided researchers with the necessary tools to assess BAT’s therapeutic potential in treating human obesity.

## MORPHOLOGICAL, FUNCTIONAL, AND GENETIC DIFFERENCES BETWEEN BAT AND WAT IN HUMANS

Compared to WAT, human BAT is highly vascularized and contains more non-medullated nerve-fibers, which gives it a deeper color. BAT also plays an important role in energy dissipation, whereas the propensity to store energy in WAT is much higher since it can store excess calories as large, intracellular triacylglycerol (TG) droplets; the fat droplets from BAT on the other hand, are much smaller and contain numerous, similar-sized locules, which are conducive to rapid oxidation of lipids (Table 1). In the course of glucose and fatty acid catabolism, the BAT marker UCP1 mediates proton uncoupling over the inner mitochondrial membrane, thereby converting fuel substrates into heat instead of ATP, a process known as non-shivering thermogenesis (Daniel Ricquier, 1976; Nicholls and Locke, 1984). However, no UCP1 expression is detected in WAT (Virtanen et al., 2009). During cold exposure, non-shivering thermogenesis stabilizes core body temperature by consuming energy via oxidative metabolism. To maintain energy levels, the glucose uptake rate of hBAT is increased by a factor of 12, while plasma insulin concentration is concurrently decreased and hWAT is unaffected (Orava et al., 2011). For comparison, insulin only enhances BAT glucose uptake 5-fold, whereas the effect on WAT is even smaller (Orava et al., 2011). The expression level of GLUT4, the insulin-responsive glucose transporter, is higher in BAT than in WAT, an observation that might underlie greater glucose uptake in BAT versus WAT (Virtanen et al., 2009). Interestingly, glucose can induce thermogenesis of hBAT in a sympathetic-independent manner (Lee et al., 2016). These results provoke the hypothesis that converting WAT into BAT is a promising approach to treat diabetes mellitus. However, the mechanisms of hBAT glucose uptake are not well understood. UCP1, the BAT marker gene, releases stored caloric energy as heat by uncoupling protons produced by oxidative phosphorylation (Cypess et al., 2009). The transcription factor PR domain containing 16 (PRDM16) is a critical switch factor in determining the commitment of mouse Myf5^+^ precursors to brown adipocytes versus myogenic cells (Jimenez-Preitner et al., 2011; Kajimura et al., 2009). PRDM16 is more highly expressed in hBAT than hWAT. Similarly, other BAT-related mRNAs such as type 2 iodothyronine deiodinase (D2), peroxisome-proliferator-activated receptor g coactivator 1α (PGC-1α), and β3-adrenergic receptor also occur at significantly higher levels in hBAT versus hWAT (Virtanen et al., 2009). These results suggest that these genes play important roles in promoting BAT development, and their function in hBAT might emulate what is observed in mice.

**Table 1:** Differences between brown and white adipocytes

	Brown	White	Ref.
Anatomical location	Thyroid, supraclavicular, mediastinal, parathoracical, perirenal	Subcutaneous, mesenteric, retroperitoneal, omental	Cypess et al. (2009), Heaton (1972)
Cellular morphology	Numerous, small lipid droplets	Unilocular, large lipid droplets	Heaton (1972)
Blood supply and nerve-fibers	Abundant	Low	Heaton (1972), Hatai (1902)
Regulatory function	Dissipating energy as heat production	Storing energy as triglycerides	Smith and Roberts (1964)
Mitochondria	Abundant	Low	Heaton (1972), Nicholls and Locke (1984)
UCP1	High	Nearly undetectable	Virtanen et al. (2009), Svensson et al. (2011)
Biomarkers	UCP1, LHX8, and ZIC1,	MPZL2, HOXC9, EBF3, FBXO31, and LEP,	Cypess et al. (2013)
Insulin sensitivity	High	Low	Orava et al. (2011)
Activators	Cold, exercise	High-calorie diet	Saito et al. (2009), Orava et al. (2011), Carriere et al. (2014)
Obesity and type 2 diabetes	Negative effect	Positive effect	Eger (1954), Himms-Hagen (1979)

## IS ADULT HUMAN BAT A CLASSICAL BAT OR BEIGE/BRITE ADIPOSE TISSUE?

Two distinct types of UCP1-positive adipocytes have been identified in mice: the classical brown fat cells and brown-like fat cells (also called beige or brite adipocytes) (Ishibashi and Seale, 2010; Seale et al., 2008). Unlike classical brown fat, which is derived from a myf-5 lineage (Seale et al., 2008), beige cells in WAT are derived from a non-myf-5 lineage and share features of both white and brown fat cells, including high expression of white adipocyte-specific markers (e.g. aP2, adipsin, and PPAR-γ) (Ishibashi and Seale, 2010). Adult hBAT, which is dispersed mainly in the supraclavicular, para-aortic, and suprarenal regions and is densely innervated, could be activated by cold through the sympathetic nervous system (Cannon and Nedergaard, 2011; Lowell and Spiegelman, 2000). However, unlike the classical brown adipocytes found in the interscapular regions of rodents and human infants, adult hBAT shares many molecular, histological, and functional characteristics with cold-induced beige fat found in the sWAT of rodents (Wu et al., 2012; Qiu et al., 2014). For example, mouse beige cells express Slc27a1, TMEM26, TBX1, CD40, and CD137 in a beige-selective manner, some of which, such as CD137, TMEM26, and TBX1, are highly expressed in adult hBAT (Wu et al., 2012), indicating that cold-induced hBAT is more closely related to rodent beige fat rather than classical brown fat. Within adult human neck BAT, the gene expression pattern of superficial neck fat resembles that of mouse sWAT, while deep neck fat is more like mouse BAT with high UCP1, PGC1-α, and D2 levels (Qiu et al., 2014). A whole-genome microarray experiment showed that the best molecular markers for grouping human fat cells are: 1) MPZL2, HOXC9, EBF3, FBXO31, and LEP, as WAT markers; 2) TNFRSF9, TMEM26, and SHOX2, as beige/brite markers; and 3) UCP1, LHX8, and ZIC1, as BAT markers (Table 1) (Cypess et al., 2013). Taken together, these studies suggest that cold-induced activation of hBAT primarily involves beige fat recruitment. However, more detailed work is needed to determine whether cold-induced activation of hBAT is mediated mainly by the sympathetic nervous system (SNS) or by alternative activation of the eosinophils-type 2 cytokine-macrophage signaling pathway (Qiu et al., 2014), or both.

## DISTINCTION OF HUMAN BAT AND MOUSE BAT

Much of our knowledge about BAT is based on mouse studies and thus, it is of great interest to compare functionality, gene, and protein expression of mouse and human BAT. However, the principal rodent BAT depot lies in a well-defined anatomical location and is homogeneously composed of brown adipocytes, whereas hBAT is widely dispersed and occurs as a mixture of white, classical brown, and recruitable brite adipocytes (Jespersen et al., 2013) (Table 2). Comparison of microRNA expression profiles between mouse and human BAT reveals that while 145 miRNAs are expressed in both species, at least 250 other miRNAs are expressed exclusively in one species or the other (Guller et al., 2015). It is important to note too that the homology between mouse and human UCP1 is less than 80%, suggesting there may be functional differences between species (Hughes et al., 2009). In addition, rodent UCP1 kinetics have been extensively studied in a variety of expression systems, whereas no corresponding study on human UCP1 kinetics has been reported to date (Hirschberg et al., 2011). Regarding thermogenic signal transduction, murine BAT is profoundly activated by selective β3 adrenergic agonists (Feldmann et al., 2009). In human studies, treatment with the β3 adrenergic agonist, mirabegron, or non-selective beta-blocker, propranolol, stimulated and impaired FDG uptake, respectively (Feldmann et al., 2009; van Baak et al., 2002). These reports suggest that β3 adrenergic receptor signaling is capable of boosting hBAT metabolic activity. Nevertheless, there have been some conflicting reports suggesting the β3 adrenergic response is very weak or non-responsive in hBAT; it has been suggested this is mainly due to sequence differences between the rodent and human receptors as well as low efficacy of the human β3-adrenergic receptor in adipose tissue and poor drug bioavailability (van Baak et al., 2002) (Table 2). A very recent study also showed that glucocorticoids can acutely increase isoprenaline-stimulated respiration and UCP1 expression in human primary brown adipocytes, but substantially decrease isoprenaline-stimulated respiration and UCP1 in mouse primary brown adipocytes (Ramage et al., 2016) (Table 2), again suggesting the presence of species-specific differences between human and mouse BAT. More prospective studies would be of great value for future delineation of the difference between human and mouse BAT biology.

**Table 2:** Differences between human and mouse adipocytes

	Human BAT	Murine BAT	Ref.
Distribution	Dispersed	The main BAT depots are in well-defined anatomic sites	Heaton (1972), Jespersen et al. (2013)
Cell composition	A mix of white, classical brown, and recruitable brite adipocytes	Homogeneously composed of brown adipocytes	Chen et al. (2013), Jespersen et al. (2013)
MicroRNA expression	145 miRNAs are expressed in BAT of both species while expression of at least 250 other miRNAs is exclusive to one species or the other		Guller et al. (2015)
Response to β3 adrenergic agonists	Weak	Strong	Feldmann et al. (2009), van Baak et al. (2002)
Changes with increasing age	Gradually decline	Stably exist	Heaton (1972), Smith and Roberts (1964)
Glucocorticoid effects	Acutely increase but chronically suppress BAT activity	Substantially suppress BAT activity	Ramage et al. (2016), Zilberfarb et al. (2001), Barclay et al. (2015)

## MECHANISTIC EFFECTS OF AMBIENT TEMPERATURE ON hBAT

Cold exposure profoundly enhances hBAT perfusion and activity, and improves glucose and fatty acid uptake, thereby supporting cold-induced energy dissipation (Saito et al., 2009; Orava et al., 2011). These observations reveal hBAT as an attractive target for treating and preventing obesity. However, the promoting effect of cold-exposure on energy expenditure is markedly suppressed by fasting-induced insulin resistance (Hanssen et al., 2015). Rodents studies show that the stimulatory effects of cold exposure are emulated by electrical stimulation of sympathetic nerves within BAT and by administration of adrenergic agonists, but abolished by surgical severing of the sympathetic nerves or adrenergic blocker treatment (Shimizu et al., 1991), indicating that cold-exposure stimulates BAT via sympathetic nerves. However, the mechanism by which the SNS regulates hBAT upon cold-exposure is still unknown. Agents that activate the SNS failed to activate BAT in human studies, presumably due to the systemic effects of SNS agonists and minimal BAT responsiveness (Cypess et al., 2012). Epinephrine, a sympathomimetic drug, does not activate hBAT at a dose that leads to broad activation of the SNS (Cypess et al., 2012), suggesting that cold-exposure activates specific sympathetic pathways responsible for hBAT activation. In addition, most known effectors of UCP1-dependent thermogenesis such as certain neuropeptides, leptin, and capsaicin all converge on SNS activation. One of the major drawbacks with using broad sympathetic activation to stimulate thermogenesis however, are the serious-side effects and increased risk for heart attack (Bonet et al., 2013). Although cold exposure profoundly increases plasma concentrations of SNS activation biomarkers like norepinephrine and the secondary messenger, cyclic adenosine monophosphate (cAMP), this effect has been found in humans both with and without cold-activated BAT (Orava et al., 2011). In addition, stimulation of the parasympathetic vagus nerve also increases energy expenditure in human BAT, although the mechanism is unclear (Vijgen et al., 2013). These reports suggest that hBAT stimulation through generalized sympathomimetic drugs is not a viable approach and thus, more extensive analyses in human BAT are needed.

Cold exposure may also alter human physiology by cross-talking with immune cells to release cytokines capable of inducing fat browning. In mice, cold exposure rapidly promoted eosinophil-dependent and interleukin 4 (IL-4)-stimulated alternative activation of adipose macrophages, which secrete catecholamines to induce thermogenic gene expression and/or lipolysis in both BAT and WAT (Qiu et al., 2014; Nguyen et al., 2011). ILC2s can promote the eosinophil/IL-4Rα/alternatively-activated macrophage (AAMac) (Qiu et al., 2014; Liu et al., 2014; Wu et al., 2011). Besides, ILC2s have been shown to promote beiging in an IL33-depend pathway in part via production of enkephalin peptides that elicit beiging (Brestoff et al., 2015). IL-4 stimulation of human monocytes has also been reported to promote alternative activation, catecholamine production, lipolytic activity of adipocytes, and dysregulated ILC2 responses in WAT are a conserved feature of obesity in humans and mice (Brestoff et al., 2015), suggesting these pathways may play the same roles across species (Nguyen et al., 2011). In humans, mild cold exposure increases plasma levels of fibroblast growth factor-21 (FGF-21) (Lee et al., 2013), a hormone that promotes systemic glucose uptake and oxidation. In hBAT, FGF-21 expression positively correlates with UCP1 expression, promotes BAT activity and recruits inducible brown adipocytes via autocrine and/or endocrine mechanisms (Hondares et al., 2014). Future studies aiming to identify secreted factors that promote thermogenesis and/or beiging will undoubtedly advance our understanding about thermogenic regulation and may lead to novel anti-obesity drug treatments.

## EFFECTS OF EXERCISE ON hBAT ACTIVITY AND THE UNDERLYING MECHANISMS

**Figure 1:**
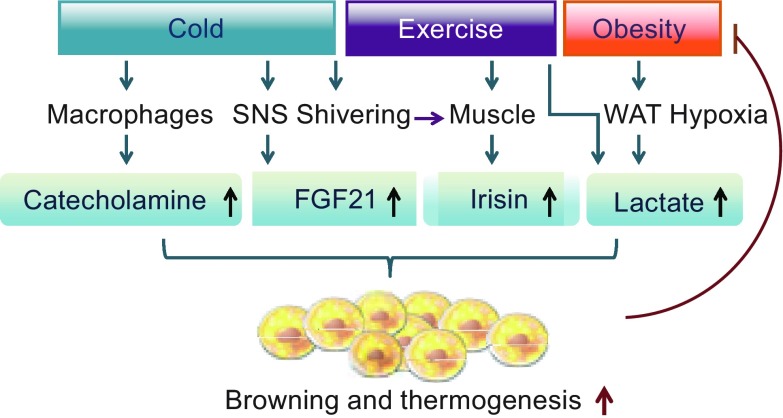
**The mechanisms by which cold, exercise, and obesity regulate thermogenesis**. Cold exposure could improve browning and thermogenesis of human adipocytes through macrophages, sympathetic nervous system (SNS), and muscle contraction. Cold exposure rapidly promoted alternative activation of adipose tissue resident macrophages, leading to the secretion of catecholamines to induce beige fat development and thermogenesis. Cold activates the SNS, leading to the upregulation of fibroblast growth factor 21 (FGF21). Cold and exercise cause muscle contraction, which stimulates irisin secretion and induces shivering thermogenesis. In response to cold-exposure, the shivering-induced irisin promotes browning and thermogenesis of human adipocytes in concert with the SNS-induced FGF21. Exercise is also able to promote browning and thermogenesis by increasing lactate production. In obese individuals, enhanced lactate in adipocytes promotes browning and thermogenesis of neighboring adipocytes, which in turn, limits the accumulation of fat, thereby impeding the development of obesity

Exercise could produce energetic benefits much greater than the direct calories cost (Speakman and Selman, 2003). Irisin, a myokine that upregulates human brown adipocyte gene expression and thermogenesis in human neck adipocytes, was found to be enhanced by exercise in humans (Bostrom et al., 2012; Lee et al., 2014). Interestingly, cold-induced shivering by muscle contraction stimulates irisin secretion to levels that are nearly comparable to those induced by exercise (Lee et al., 2014), suggesting that irisin may link exercise to cold-induced thermogenesis and promote browning of human neck adipocytes and thermogenesis in concert with FGF21 during cold-exposure (Lee et al., 2014) (Fig. 1). However, another study showed that neither acute nor regular exercise altered irisin levels, although the intensity of habitual physical activity, muscle volume, strength, contractility are positively associated with circulating irisin (Kurdiova et al., 2014). Thus, it remains to be further determined as to whether irisin regulates thermogenesis in humans. Besides irisin, increased plasma lactate concentrations resulting from strenuous exercise may also contribute to browning. Lactate, which is a glycolytic waste product, drives the browning of human white adipocytes through drastic and rapid inductions of several brown-adipocyte genes including UCP1 via peroxisome proliferator activated receptor-γ (PPAR-γ), a master transcription factor regulating adipocyte differentiation (Carriere et al., 2014). Furthermore, obesity elevates lactate production in rat WAT due to hypoxia (DiGirolamo et al., 1992). The lactate then stimulates “browning” of neighboring white adipocytes through an autocrine/paracrine effect. This could reflect a self-regulatory response to limit the accumulation of fat during obesity (Trayhurn and Alomar, 2015). Indeed, fat cells cultured from obese or diabetic humans metabolize more lactate than lean individuals (DiGirolamo et al., 1992), which indicates that lactate might play the same role *in vivo*. Human natriuretic peptides (NPs) also play a physiological role in exercise-induced lipolysis (Moro et al., 2008). In human adipocytes, atrial natriuretic peptide (ANP) and brain natriuretic peptide (BNP) induce a brown-like adaptive thermogenic response by activating PPARγ coactivator-1α (PGC-1α) and UCP1 expression and stimulating mitochondrial biogenesis, thereby increasing uncoupled and total respiration (Bordicchia et al., 2012). Beyond the calories burned at the gym, it is becoming increasingly clear that changes in both gene expression and the secretome are likely responsible for at least some of the beneficial effects of exercise on whole body energy expenditure (Speakman and Selman, 2003).

## REGULATORY FACTORS OF HUMAN BAT

Human brown adipocytes are regulated by many intrinsic factors, including glucocorticoids and bile acids. In the absence of glucocorticoids, brown adipocytes differentiate poorly, particularly during early differentiation. Glucocorticoids induce PPAR-γ and CCAAT/enhancer binding protein-β (C/EBP-β) expression as well as increase PGC1-α and GLUT-4 levels, while reducing the expression of DNA binding-2 inhibitor (Id2) and TNF-α (Ramage et al., 2016; Zilberfarb et al., 2001). Acute treatment of human brown adipocytes with glucocorticoids at physiological concentrations increased basal and adrenergic stimulated respiration and UCP1 expression (Ramage et al., 2016). However, chronic glucocorticoid stimulation also suppressed β-adrenergic thermogenesis in hBAT (Ramage et al., 2016; Barclay et al., 2015), which may underlie glucocorticoid therapy-associated body-weight gain. The bile acid, chenodeoxycholic acid (CDCA), is another steroid molecule that is essential for metabolism and thought to increase human whole-body energy expenditure. In human brown adipocytes, CDCA improves mitochondrial uncoupling and D2 expression via a G protein-coupled receptor TGR5 (G-protein coupled receptor for bile acids)-dependent mechanism (Broeders et al., 2015). As a registered and generally recognized safe drug for humans (Broeders et al., 2015), CDCA is a promising candidate to activate hBAT and counter obesity and its related metabolic diseases. Thus, identification of more endogenous regulatory factors is critically needed to elucidate hBAT biology and the body’s complex responses to regulate energy expenditure.

## DEVELOPMENT OF IMMORTALIZED HUMAN BROWN ADIPOCYTES

Despite advances in our understanding of murine BAT, there remain numerous knowledge gaps concerning the biological mechanisms of hBAT. Although thermal imaging and FDG/MRI scans are capable of capturing human BAT activity non-invasively, for *in vitro* research, human cell lines are still needed to uncover the genetic, pharmacological, and environmental determinants of BAT biological functions. PAZ6, the first available immortalized human BAT cell line (Zilberfarb et al., 1997), has been used to study generic cellular processes and evaluate the expression of adipogenic markers. Differentiated PAZ6 adipocytes express a myriad of brown adipocyte markers including β1, β2, and β3 adrenergic receptors (β-AR), α2A-AR, lipoprotein lipase, hormone sensitive lipase, adipsin, the glucose transporters Glut 1 and Glut 4, leptin, and UCP1 (Zilberfarb et al., 1997). Among these BAT markers, the expression of leptin and GLUT1 is markedly stimulated by hypoxia (Grosfeld et al., 2002), which could be an adaptive mechanism that promotes angiogenesis and increases adipose tissue oxygenation (Grosfeld et al., 2002). While it is widely accepted that the rodent β3-adrenoceptor is essential for regulating fat metabolism, its role in human BAT is still under debate (Arch and Wilson, 1996). According to a saturation binding analysis using PAZ6 adipocytes, the β3-AR appears to be the most abundant β-AR subtype. The selective β3-AR agonist, CGP 12177A, markedly increases cAMP concentration and lipolysis activity in PAZ6 adipocytes (Zilberfarb et al., 1997). Pre-treating PAZ6 adipocytes with norepinephrine however, down-regulates β-AR number and desensitizes β3-AR signaling (Jockers et al., 1998). These results suggest that β3-AR in human brown adipocyte is functionally coupled to lipolysis (Jockers et al., 1998). Thiazolidinediones are a popular class of drugs used to treat type 2 diabetes because of their ability to increase insulin sensitivity by binding to PPAR-γ, which alters the expression of genes involved in glucose and lipid metabolism. PAZ6 cells treated with thiazolidinediones exhibited enhanced brown adipocyte differentiation as demonstrated by increased triglyceride content and adipocyte-specific gene expression, including aP2, PPAR-γ, β3-AR, HSL, and UCP2 (Strobel et al., 1999). Other small molecules thought to promote hBAT function such as dehydroepiandrosterone (DHEA) and retinoic acid (RA) have also been tested in PAZ6 cells. Serum concentrations of DHEA, a steroid sex hormone precursor, inversely correlate with biomarkers of metabolic syndrome, indicating that DHEA may modulate adipose tissue mass and function. DHEA inhibits PAZ6 preadipocyte proliferation by blocking the G_1_/S or G_2_/M transition. By contrast, UCP1 mRNA levels are higher in PAZ6 cells differentiated in the presence of DHEA. The inhibitory effect on PAZ6 pre-adipocyte cell cycle progression and the promoting effect on PAZ6 adipocyte gene expression may represent a pro-adipogenic mechanism (Rice et al., 2010). In PAZ6 adipocytes, retinoic acid increases UCP1 gene expression in a PGC1α-dependent way, while the PPARα-specific agonist, WY14643, failed to regulate UCP1 gene expression without RA (Oberkofler et al., 2002). However, the mechanism by which RA regulates hBAT *in vivo* remains uncertain.

## SUMMARY AND FUTURE PERSPECTIVES

The therapeutic potential of hBAT as an anti-glycemic, anti-lipidemic, and weight loss-inducing ‘metabolic panacea’ is postulated by calculations showing that when fully activated, 63 grams of BAT would burn the energy-equivalence of 4.1 kilograms of WAT over the course of a year (Virtanen et al., 2009). Considering its striking capacity for energy dissipation, the findings in hBAT are clinically valuable for guiding future prevention strategies and treatments for obesity, insulin resistance, and other related metabolic diseases (Hall et al., 2011). Increasing human energy expenditure through hBAT by cold-exposure (Cypess et al., 2009; Aherne and Hull, 1966) and exercise (Lee et al., 2013; Hondares et al., 2014) however, would require drastic changes in life style. Considering the discomfort of cold exposure, exercise is likely the preferred approach for enhancing hBAT activity. The increased irisin and lactate concentrations that are induced during exercise have been reported to promote adipocyte browning remodeling and are potential approaches for treating obesity without the exercise (Bostrom et al., 2012; Carriere et al., 2014). Molecules such as FGF-21, natriuretic peptides, and thyroid hormones have also been suggested as potential drugs to counteract obesity and its related metabolic diseases. The thyroid hormone, triiodothyronine (T3), has been shown to profoundly promote mitochondrial biogenesis and UCP1 expression in brown-like fat cells as well as skeletal muscle and brown adipose tissue (Crisan et al., 2008). Another anti-obesity strategy that has not been considered in this review, but warrant serious investigation, includes transforming muscles cells into brown fat. Interestingly, a stationary population of human skeletal muscle cells expressing the CD34 surface protein have been found to differentiate *in vitro* into genuine brown adipocytes with a high level of UCP1 expression (Crisan et al., 2008), suggesting that human skeletal muscle cells have the potential to transdifferentiate into brown adipocytes. Thus, finding new ways to reprogram skeletal muscle into brown fat represents another novel approach to promote thermogenesis and combat obesity.

Although pharmacological means to treat obesity are possible, supraphysiological concentrations of these medications are associated with severe side effects. In addition, only a few defects in human BAT development and thermogenesis due to gene mutations have been described (Yoneshiro et al., 2005) thus, a better understanding of the physiological processes underlying hBAT is still required before BAT-related therapies can be developed. At present, much of our knowledge about BAT is based on mouse studies and hBAT research is still limited by a number of factors such as access to high-quality, ethically-approved clinical samples, reliable methods for measuring BAT volume and activity, and better ways to stimulate BAT function with minimal adverse effects. One protocol, which collects both MRI and 18F-FDG PET-CT scans, has been reported to give accurate estimates of hBAT imaging volumes (Gifford et al., 2015). In the future, this approach could be used in longitudinal studies to assess therapeutic interventions. In conclusion, we opine that a better understanding of hBAT physiology will lead to new pharmacological targets for treating obesity, and given the current obesity epidemic, more physiological studies of brown adipose tissue in healthy adult individuals are needed to fully characterize its regulatory function and therapeutic potential.
